# Efficacy of Oral Valacyclovir Treatment in a Case with Acute Retinal Necrosis

**DOI:** 10.4274/tjo.14890

**Published:** 2017-06-01

**Authors:** Mert Şimşek, Pınar Çakar Özdal, Kemal Tekin

**Affiliations:** 1 Ulucanlar Eye Training and Research Hospital, Ophthalmology Clinic, Ankara, Turkey

**Keywords:** Acute retinal necrosis, treatment, valacyclovir, prognosis

## Abstract

Acute retinal necrosis (ARN) is a rapidly progressive disease with poor prognosis, leading to visual loss in most cases. Rapid diagnosis and early anti-viral treatment significantly affect the course and prognosis of the disease. In this case report, we present a 34-year-old female patient referred to our clinic with symptoms of blurred vision and ocular pain diagnosed as acute glaucoma elsewhere. A clinical diagnosis of ARN was made and anti-viral treatment was started immediately. We herein describe our treatment approach to this particular case and discuss previously reported treatment modalities.

## INTRODUCTION

Acute retinal necrosis (ARN), first described in 1971, is a clinical condition characterized by areas of retinal necrosis, occlusive vasculopathy, vitritis, anterior chamber reaction, and optic neuritis.^1,2,3,4^ Herpes virus family members herpes simplex virus (HSV), varicella zoster virus (VZV), Epstein-Barr virus, and cytomegalovirus play a role in its etiology.^2,3,4,5,6^ The most common agent is reported as VZV in some studies and HSV-1 in others.^5^ Ganatra et al.^6^ reported that VSV and HSV-1 were more common in patients over 25 years old, while HSV-2 was more common in those under 25 years old.

Prompt intervention is very important after ARN is detected. Patients with delayed treatment suffer rapidly progressive retinal necrosis; exudative, rhegmatogenous, or tractional detachment may occur, with possible outcomes as severe as phthisis bulbi. Treatment delay of more than 14 days after symptom onset has been reported as one of the factors associated with poor prognosis.^5^ There is no standard treatment approach due to the rare occurrence of ARN and the fact that the data available in the literature consists of small case series. There are studies reporting favorable outcomes with early systemic antiviral therapy, intravitreal injections, and early vitrectomy.^7,8^

The most commonly used and current gold standard initial treatment for ARN is acyclovir; other antiviral options including valacyclovir, famciclovir, ganciclovir, valganciclovir, and foscarnet are also used.

Acyclovir is an antiviral guanosine analogue with proven efficacy against various viral agents, primarily HSV infections. It prevents viral replication by inhibiting viral DNA polymerase and may be administered via oral and intravenous systemic routes.^9^

Valacyclovir is another antiviral drug with proven efficacy against HSVs. Valacyclovir is a prodrug which is converted in vivo via hepatic first-pass metabolism to acyclovir, which is then modified by viral thymidine kinase and prevents viral proliferation.^9^ In recent years, it has been reported that oral valacyclovir and intravenous acyclovir yield comparable results.^10^

In this report, we share a case of acute retinal necrosis that was completely controlled with oral valacyclovir therapy and discuss ARN treatment.

## CASE REPORT

**Figure 1:**
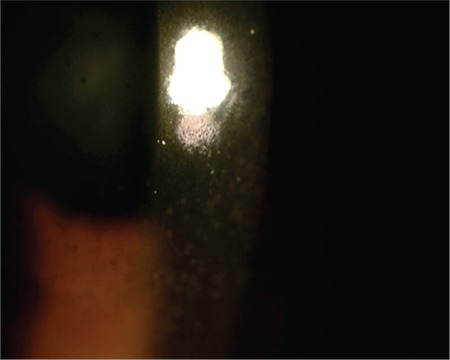
Diffuse brownish-gray, medium-sized keratic precipitates in the corneal endothelium

**Figure 2:**
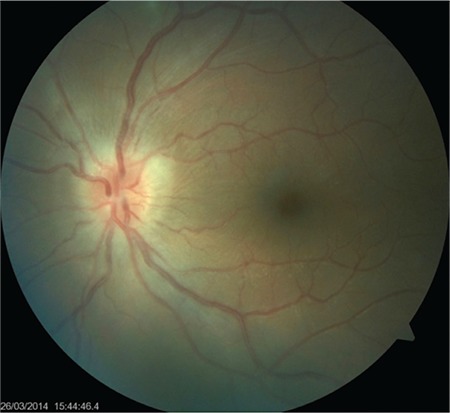
Blurred margins, hyperemia, and swelling of the optic disc

**Figure 3:**
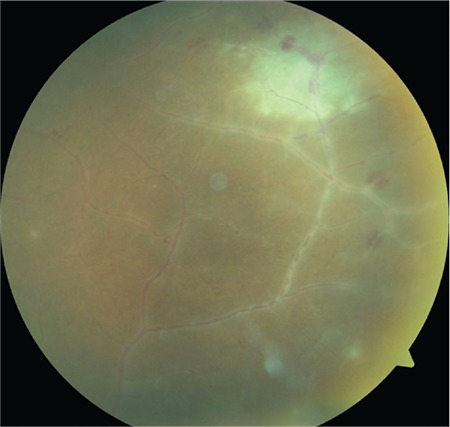
Peripheral vascular sheathing associated with vasculitis and a focus of hemorrhagic necrotizing retinitis in the superotemporal peripheral retina

**Figure 4:**
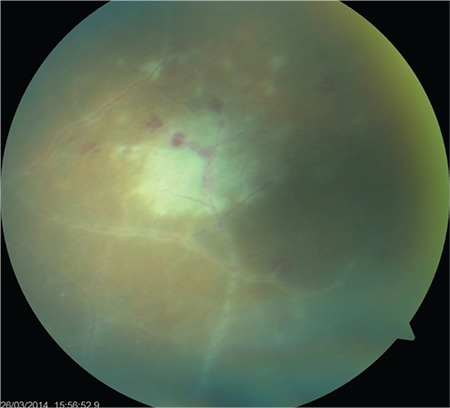
Numerous new infiltrates at the periphery of the necrotizing retinitis focus. Untreated, they progress by enlarging and merging

A 24-year-old female patient presented to our clinic with complaints of blurred vision and pain in her left eye for 4 days. She reported that at another medical center, her intraocular pressure had been measured as 38 mmHg and she had been treated with intravenous mannitol, oral acetazolamide, and topical antiglaucomatous therapy for glaucoma. On ophthalmologic examination, her BCVA was 10/10 in the right eye and 5/10 in the left eye. Intraocular pressure was 12 mmHg in the right eye and 14 mmHg in the left eye. Examination findings were normal in the right eye. In the left eye, diffuse, medium-sized brownish-gray keratic precipitates (KP) were observed in the corneal endothelium (Figure 1) and 2+ cells were noted in the anterior chamber. Dilated fundus examination of the left eye revealed a small amount of vitreous cells and 1+ haze. There was pronounced hyperemia and swelling of the optic disc (Figure 2). There was peripheral vascular sheathing associated with vasculitis and a focus of hemorrhagic necrotizing retinitis in the superotemporal periphery (Figure 3), with multiple smaller foci located more peripherally (Figure 4). The patient reported no systemic symptoms or history of systemic disease.

The patient was diagnosed with ARN based on clinical findings and treatment with prednisolone acetate drops hourly, cyclopentolate hydrochloride drops 3 times daily, and intravenous acyclovir 750 mg, 3 times daily was initiated for anterior segment inflammation. Fundus fluorescein angiography (FFA) examination was recommended to confirm clinical findings, but the patient did not consent to the procedure. On day 3 of treatment, the patient decided she did not want to remain hospitalized and undergo the 14-day intravenous acyclovir treatment plan, and her therapy was changed to oral acyclovir 2 g, 3 times daily. On examination performed that day, the patient’s corrected visual acuity was 5/10 in the left eye and fundus examination revealed no further progression of the retinal lesions. On day 4 of treatment, oral methylprednisolone 64 mg was added for optic neuropathy and severe vasculitis. The patient was followed closely at intervals of 2 days.

On day 7 of treatment, the patient’s corrected visual acuity was 8/10. The KP and vitreous haze persisted, but fundus examination revealed no progression of the retinal lesions, reduced optic disc edema, and more distinct optic disc margins.

On day 9, the patient’s corrected visual acuity was 10/10. On fundus examination, +1 vitreous haze and regression of optic disc edema were observed. The KP were still evident. Treatment was adjusted to prednisolone acetate drops every 2 hours and cyclopentolate hydrochloride twice daily; oral valacyclovir 2 g, 3 times daily was continued. Methylprednisolone was reduced to 54 mg.

**Figure 5:**
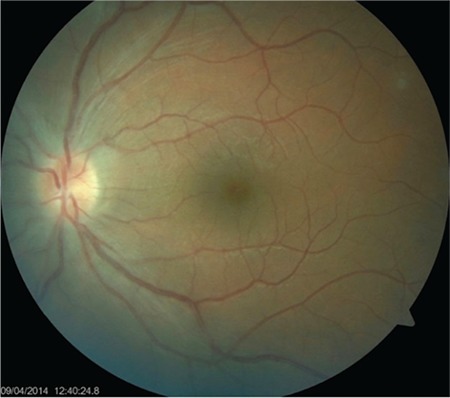
Retinal appearance on day 14 of treatment; optic disc findings have improved

**Figure 6:**
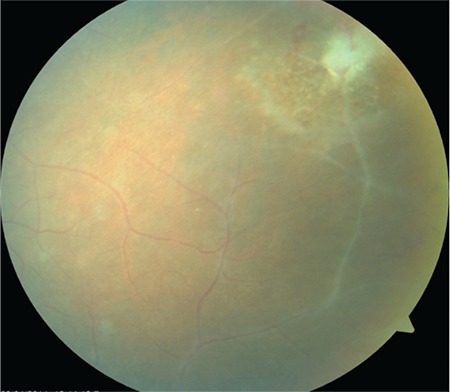
The focus of necrotizing retinitis reduced in size and regressed; empty vessels are apparent

On day 14 of treatment, visual acuity was 10/10, KP had resolved, complete resolution of the vitreous haze and optic disc signs was observed on fundus examination, and the necrotizing retinitis focus had regressed (Figures 5 and 6). Prednisolone acetate was reduced to 5 times daily, cyclopentolate hydrochloride to once daily. Valacyclovir dose was adjusted to 1 g, 4 times daily and methylprednisolone to 48 mg, planning to reduce the dose by 8 mg every 3 days.

On day 22 of treatment, the lesioned area of the retina appeared extremely atrophic and 3 rows of prophylactic laser photocoagulation was applied to this area only.

On day 35 of treatment, fundus examination revealed empty vessels in the peripheral retina and the lesion had completely resolved. Valacyclovir therapy was maintained at 1 g, 3 times daily, while the local treatment and methylprednisolone were discontinued.

At 2 months after the initiation of treatment, the dose of oral valacyclovir was adjusted to 1 g twice daily, with a plan to reduce it by 500 mg with monthly follow-up examinations.

The patient regularly attended monthly follow-up, and was maintained on oral valacyclovir 500 mg daily from 6 months to 1 year.

**Figure 7:**
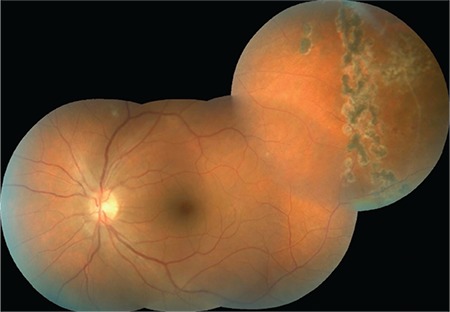
Retinal appearance after 1 year; laser scars are evident over the inactive necrotic retinal focus

At 1 year of treatment, corrected visual acuity was 10/10, KP had disappeared, the optic disc appeared normal on fundus examination, and pigmented laser scars were evident over the empty vessels in the peripheral retina and the inactive superotemporal necrotic retinal focus (Figure 7).

## DISCUSSION

ARN is a syndrome caused by herpes viruses and does not show age or sex differences. In 1994, the American Uveitis Committee defined the criteria for this syndrome as follows:

- One or more areas of retinal necrosis in the peripheral fundus,

- Rapid progression in the absence of antiviral therapy,

- Circumferential spread,

- Occlusive vasculopathy with arterial involvement,

- Inflammatory reaction in the anterior chamber and vitreous.

Patients with ARN frequently present with unilateral pain, photophobia, redness, blurred vision, and floaters. On examination, one or more of the following findings may be observed in the anterior segment: episcleritis, scleritis, keratitis, anterior chamber cells, keratic precipitation (granulomatous/non-granulomatous). In the posterior segment, signs of vitreous inflammation (haze, cells, etc.), foci of retinal necrosis, vascular sheathing, areas of hemorrhage consistent with the vessel walls, and optic neuropathy characterized by optic disc edema and margin obscuration may be observed.^2,3^

Although ARN is usually a clinical diagnosis, analysis of anterior chamber and vitreous specimens may facilitate definitive diagnosis in uncertain cases.^2^

Due to its rare occurrence and lack of large case series, there is no established standard treatment scheme for ARN. Therefore, treatment may vary between medical centers. Intravenous acyclovir is the most commonly used therapy because of its high bioavailability. There are also different approaches regarding treatment duration. The most commonly recommended regimen is 10-21 days intravenous acyclovir therapy followed by at least 6 weeks of oral acyclovir.^11,12^ However, some authors assert that longer maintenance therapy is necessary.

In addition, there are also studies recommending oral valacyclovir and famciclovir, or intravenous foscarnet and gancyclovir as alternatives.^11,12^

Valacyclovir is a L-valine esterified prodrug of acyclovir. After passing through the intestine, it is converted to an active form via hepatic first-pass metabolism.^9^ Previous studies have shown that valacyclovir has better bioavailability than acyclovir and results in higher serum levels compared to acyclovir administration.^13,14^

Taylor et al.^10^ reported first treatment response after an average of 7 days and complete resolution of retinitis after an average of 21 days of treatment in 10 ARN eyes treated with oral valcyclovir 2 g, 3 times daily. They emphasized that outcomes with oral valacyclovir were comparable to those achieved with intravenous acyclovir, with no recurrence or fellow eye involvement. Among our patients, first treatment response was noted after 9 days of treatment and complete resolution after 14 days.

ARN is usually seen in immunocompetent individuals, first appearing in one eye and later affecting the fellow eye in approximately one-third of cases. Fellow eye involvement usually appears within the first 6 weeks, although it has also been reported to develop months or even years later.^15,16^ We did not observe fellow eye involvement in our patient during the 16-month follow-up period. The long-term maintenance and gradual tapering of valacyclovir therapy has an important role in this process. In a retrospective analysis of ARN cases, Palay et al.^17^ reported that the group maintained on prophylactic antiviral therapy for 12 months had significantly better protection of the fellow eye compared to the group whose antiviral therapy was discontinued. In our case, oral valacyclovir therapy was initiated at 2 g, 3 times daily. The dose was reduced gradually according to disease course with regular follow-up examinations. We tapered the oral valacyclovir therapy to 500 mg after 6 months of treatment and maintained this dose for an additional 6 months to prevent fellow eye involvement.

Chen et al.^18^ reported that oral valacyclovir therapy was effective in a case with multiviral infection, but the patient’s final visual acuity was low due to macular detachment after therapy was discontinued. The multiviral infection in the etiology of that case suggests that the patient may have been immunosuppressed, and that valacyclovir therapy may be less effective in such cases than in immunocompetent patients.^19^

It is recommended to add systemic corticosteroids to treatment in addition to antiviral therapy in patients with ARN, especially in cases with optic neuropathy and to suppress inflammation.^2,11,20^ It is critical to initiate systemic steroid therapy after antiviral therapy, and to discontinue it before antiviral therapy is discontinued. Otherwise, it is known to lead to viral replication.^20^ A very recent study has demonstrated that ARN patients previously treated with systemic corticosteroids alone for various diagnoses had a longer healing time (mean: 53.8 days) compared to those who were not treated with corticosteroids (mean: 32.5 days).^5^ We believe that the corticosteroids we administered within the antiviral therapy regimen was instrumental in the regression of inflammatory signs such as optic neuropathy, vascular sheathing, and vitreous haze in our patient.

There is debate in the literature regarding the place of prophylactic photocoagulation therapy in ARN. Lau et al.^2^ showed that photocoagulation performed in the first 2 weeks of ARN reduced the risk of detachment. However, Tibbetts et al.^11^ found that laser therapy conferred no additional advantage and even reported increased detachment rates in patients that underwent laser therapy.

Despite good treatment response in our patient, we decided to perform prophylactic laser photocoagulation on the area of necrosis and atrophic retinal focus in the superotemporal quadrant in order to reduce the likelihood of rhegmatogenous detachment.

The place of early vitrectomy in ARN treatment is also controversial. Hillenkamp et al.^19^ reported that early vitrectomy had no effect on functional outcomes, but lowered the risk of secondary detachment. Regardless, vitrectomy is unavoidable in cases that develop complications like vitreous hemorrhage or retinal detachment.

In clinical practice, prompt hospitalization with rapid initiation of antiviral therapy in patients diagnosed with this syndrome is the main factor determining future visual prognosis.^8^ Factors strongly affecting prognosis are time from symptom onset to diagnosis (better prognosis if less than 2 weeks), extent of retinal lesions, and presence of macular involvement.^2,6^ Considering those factors, in addition to early diagnosis and treatment, close monitoring of treatment response and disease progression also substantially contributed to the complete recovery of our patient’s visual acuity. Examinations were performed daily for the first 3 days of treatment, then every other day until a full response was observed at 2 weeks.

Many retinal syndromes and diseases may manifest with similar fundus appearance. The exclusion of other conditions is essential for reaching a definitive diagnosis and initiating appropriate treatment rapidly and effectively.

Although diagnosis of ARN is based on clinical observations and lesion progression according to the criteria defined by the American Uveitis Committee, additional diagnostic methods are required to exclude similar clinical presentations and confirm the diagnosis. The most important of these is viral DNA detection from anterior chamber fluid or vitreous samples via polymerase chain reaction analysis.^2,21^ Although not directly diagnostic, FFA may be beneficial to support clinical findings in early cases without severe vitreous haze. In our case, we did not take an anterior chamber fluid sample to confirm our clinical diagnosis, and we could not perform FFA because the patient did not consent to the procedure.

In summary, patients with ARN should be diagnosed carefully, treated promptly, and followed closely. ARN should come to mind for patients presenting with a clinical constellation of unilateral, brownish-gray KP, acute intraocular pressure elevation, and optic neuritis, and a detailed peripheral retinal examination should be performed in such cases. The possibility of fellow eye involvement must be remembered during follow-up, and antiviral therapy should be maintained over the long term. High-dose oral valacyclovir therapy should be considered as an alternative to intravenous acyclovir therapy.
